# Reducing the Risks of Esophagectomies: A Retrospective Comparison of Hybrid versus Full-Robotic-Assisted Minimally Invasive Esophagectomy (RAMIE) Approaches

**DOI:** 10.3390/jcm12185823

**Published:** 2023-09-07

**Authors:** Jens Peter Hoelzen, Brooke E. Frankauer, Carsten Szardenings, Dhruvajyoti Roy, Lukas Pollmann, Lukas Fortmann, Jennifer Merten, Emile Rijcken, Mazen A. Juratli, Andreas Pascher

**Affiliations:** 1 Department of General, Visceral and Transplant Surgery, University Hospital Muenster, 48149 Muenster, Germany; brooke.frankauer@ukmuenster.de (B.E.F.); andreas.pascher@ukmuenster.de (A.P.); 2 Institute of Biostatistics and Clinical Research, University of Muenster, 48149 Muenster, Germany; 3 Department of Surgical Oncology, The University of Texas MD Anderson Cancer Center, Houston, TX 77030, USA

**Keywords:** esophageal cancer, hybrid-RAMIE, full-RAMIE, Ivor Lewis esophagectomy, perioperative outcomes, morbidity, risk mitigation

## Abstract

This retrospective analysis aimed to assess and compare the short-term perioperative outcomes and morbidity of hybrid and full-Robotic-Assisted Minimally Invasive Esophagectomy (RAMIE) surgical techniques. A total of 168 robotic-assisted Ivor Lewis esophagectomy procedures performed at Muenster University Hospital were included in the study, with 63 cases in the hybrid group and 105 cases in the full-robotic group. Demographic factors, comorbidities, and tumor stages showed no significant differences between the two groups. However, the full-RAMIE technique demonstrated superiority in terms of overall operative time, postoperative pain levels, and patient morphine consumption. Additionally, the full-RAMIE group exhibited better perioperative outcomes, with significantly shorter ICU stays and fewer occurrences of pneumonias and severe complications. While there was a trend favoring the full-RAMIE technique in terms of severe postoperative complications and anastomotic insufficiencies, further research is required to establish it as the gold standard surgical technique for Ivor Lewis esophagectomy.

## 1. Introduction

Esophageal cancer is the eighth most common type of cancer worldwide and the sixth leading cause of cancer deaths [1]. Despite the expansion of preventive measures and a heightened medical and societal awareness of risk factors (i.e., nutrition, alcohol, smoking, GERD), the incidence of esophageal cancer is “expected to increase by roughly 35% from 2018 to 2030” [2], thus making it one of the fastest growing malignancies. With an approximate 40% five-year survival rate, esophageal cancer also remains one of the deadliest cancers perioperatively. However, advances in surgical–oncological care have the potential to lead to higher survival rates and better long-term treatment options [3]. To this end, it is not just the pathology itself that remains challenging: esophagectomy as a surgical procedure suffers from an array of varying routine approaches (open, minimally invasive, hybrid or full robotic) and lack of a clear gold standard technique. Ultimately, this plurality leads to a more complex assessment of perioperative outcomes, and as such, imminently suboptimal disease management.

While it is clear that surgical resection with neoadjuvant therapy continues to be the optimal treatment as it provides potential curative outcomes, the extent of resection and ideal surgical approach remain contentious, and consequently varies greatly between institutions [4,5,6]. The growing adoption of robotic-assisted minimally invasive esophagectomy (RAMIE) presents one pillar of a safe surgical resection that yields significant short-term benefits over other hybrid minimally invasive esophagectomy (MIE) and open surgery (OE) techniques, such as reduced hospital stays, faster functional recovery, lower postoperative pain, and fewer overall severe complications [7,8,9,10,11]. 

Historically, esophagectomy procedures have widely been recognized as one of the most complex major oncologic operations [12]. MIE approaches have been shown to reduce surgical trauma, pain, and postoperative complications, thus affecting both short-term and long-term quality of life [13,14,15]. Reduced peri- and postoperative complications have allowed RAMIE to emerge not only as a desired treatment option for patients but also as an optimal solution to the technical limitations of conventional MIE, namely the use of rigid, straight instruments that have a large distance between trocar and the target anatomy [9,10,16]. Furthermore, RAMIE is associated with an increased rate of textbook outcomes that could prevent postoperative complications and improve long-term survival [17,18]. 

These factors support the clear trend in the literature towards increasingly minimally invasive procedures, which favors RAMIE to MIE. Although RAMIE has additional benefits such as better visualization, ability to perform more complex surgical maneuvers, and more precise lymph node dissections in smaller spaces [19,20,21,22,23], the evidence for robotic use in the abdominal phase is minimal [24,25]. With continuous advancement in the technological capabilities of robotic software between the Si and Xi da Vinci Robot systems (Da Vinci Robotic System Intuitive Surgical Inc., Sunnyvale, CA, USA), machine learning, and surgeon experience, the RAMIE method has an ever-growing list of potential benefits to patients, making robotic dissection in tight spaces overall superior [9,23,26]. It is this notable innovation in robotic technology that now allows for a full-robotic approach. It is also what motivated this retrospective study. 

Initially, our center performed a hybrid-RAMIE technique with conventional abdominal laparoscopy and robot-assisted thoracic phase using the Si da Vinci robot system. With the implementation of the Xi da Vinci robot system, the minimally invasive technique went a step further. The ability to use Endowrist instruments and the smaller robotic arms made multiquadrant surgery in the abdominal phase possible [24]. The trajectory of RAMIE approaches at our center highlights an inherent issue: although gradual advancements in technology allow for what we can safely, if cautiously, call a potential refinement of techniques, the procedure’s nuanced spectrum and limited (yet growing) availability of new robotic systems means that a surgical gold standard for RAMIE techniques has yet to be established [5]. It is from these considerations that this study aims to compare the two most commonly performed RAMIE techniques, and in doing so, hopes to make a valuable contribution not only to the field of upper gastrointestinal (robotic) surgery, but also to the perioperative care, treatment, and outcomes of esophagectomy patients. As a practical and patient-centered way forward, we hope that our findings lead to the establishment of a gold standard technique, and yet more effectively, the widespread adoption of the full-RAMIE procedure. 

## 2. Materials and Methods

### 2.1. Study Design

This study is a university single-center, investigator-initiated, retrospective comparison efficacy trial of robot-assisted hybrid-RAMIE and full-RAMIE surgical techniques in patients with resectable intrathoracic esophageal cancer. The Muenster University Hospital is a high-volume esophagectomy center with experience in OE, MIE, and RAMIE surgical techniques and therefore optimally positioned to report on short-term postoperative findings. Consequently, the main objective was to determine the efficacy of two RAMIE surgical approaches regarding short-term perioperative parameters including operating time, blood loss, incidence of severe postoperative complications (Clavien-Dindo Classification > 3b), length of hospital stay, length of Intensive Care Unit (ICU) stay, postoperative incidence of pneumonia, and anastomosis insufficiency, as defined by the Esophageal Complications Consensus Group (ECCG) [27]. Secondary endpoints include median opioid consumption in Morphine milligram Equivalent (MME), and median postoperative pain NRS (numeric rating scale).

### 2.2. Patients

Inclusion criteria were as follows: all adult patients (≥18 years) with histologically proven, surgically resectable (at the time of diagnosis) intrathoracic or abdominal esophageal carcinoma treated surgically at the Muenster University Hospital between January 2019 and December 2022. Selection between groups was established retrospectively and based on the RAMIE technique performed at the time of surgery. The hybrid-RAMIE procedures using the da Vinci Si were performed from January 2019 until December 2020, whereas the full-RAMIE procedures using the da Vinci Xi were performed from January 2021 through December 2022. All patients with cervical esophageal carcinoma, patients with carcinoma of the gastro-esophageal junction with gastric cardia infiltration (Siewert Type-III), and all patients whose surgery was intraoperatively terminated upon diagnosis of inoperability, were excluded from this study. All patients were treated with a neoadjuvant therapy, either radiochemotherapy or chemotherapy, following either the CROSS- (Carboplatin/Paclitaxel) or FLOT- schemes (Fluorouracil/Leucovorin/Oxaliplatin/Docetaxel) [4,28,29,30]. Optimal treatment was decided individually on recommendation from an interdisciplinary tumor conference board and in accordance with the guidelines set forth by the German Cancer Society (DKG). All data were gathered retrospectively from electronic patient charts and medical records. Prior to surgery, all patients received standard diagnostic care which included an esophagogastrodudodenoscopy, abdominal ultrasound, and pulmonary function test. All patients in this study were treated by the same, highly experienced surgeon, who has been performing upper gastrointestinal tract surgeries at our high-volume center since 2008. To ensure adequate perioperative pain management, each patient received an epidural catheter that was placed prior to surgery. Lastly, each patient was given intravenous antibiotics (3 g Cefuroxime and 500 mg Metronidazole) as a preoperative prophylaxis thirty minutes prior to the start of surgery.

### 2.3. Surgical Technique

The robotic-assisted Ivor Lewis minimally invasive esophagectomy (RAMIE) consists of two phases: abdominal and thoracic, with gastrolysis, en-bloc esophagectomy with a D2 lymphadenectomy and gastric conduit formation via a 45 linear gastric stapler [8,20,24,26,31]. The anastomosis perfusion is evaluated using ICG fluorescence imaging. The thoracic phase is robotic-assisted and identical in both approaches. It is only in the abdominal phase that the two groups and procedures differ. Additionally, all patients received preemptive endoluminal vacuum therapy (EndoVAC) placement under endoscopic control. Finally, it is worth pointing out our nomenclature for the surgical techniques, as what has previously been referred to in other publications as “RAMIE”, we define here as “hybrid-RAMIE”, whereas the abdominal phase is performed via conventional laparoscopy and only the thoracic phase assisted by the da Vinci Si System. In the full-RAMIE group, both thoracic and abdominal phases are robotically performed using the da Vinci Xi system.

### 2.4. Postoperative Care

Postoperative patients were first transferred to the ICU and then, once hemodynamically and respiratorily stable, moved to the general surgical ward. Physical therapy and rehabilitation were the same in both groups and included early mobilization. Patient oral intake and nutrition began with liquids and increased gradually to soft food within three postoperative days. Patients received multimodal pain therapy including epidural catheter, non-steroid anti-inflammatory drugs, Metamizol and opioid analgesics, as needed for breakthrough pain [32]. Quality of the epidural anesthesia was controlled daily by experienced acute pain specialists. On average, epidural catheters were removed on the fifth postoperative day. Only two patients did not have an epidural catheter, both of which belonged to the full-RAMIE group. Evaluation of subjective pain levels occurred routinely multiple times per day using the NRS-11 for pain. On the fifth postoperative day, a routine esophagogastroduodenoscopy (EGD) was performed to ensure anastomotic sufficiency. If irregularities were found, EndoVAC therapy was continued. Otherwise, the EndoVAC sponge was removed and a gastrin esophagogram was performed. Chest tube and wound drains were removed as soon as the drainage output was at or below 200 mL per day. Throughout the study, there were no significant changes in postoperative care or pain management. 

### 2.5. Endpoints

To compare the extent of robotic assistance in RAMIE techniques, we assessed perioperative and short-term postoperative parameters retrospectively using patient charts and data. Perioperative primary endpoints included baseline demographics such as age, sex, body mass index, Charlson-Comorbidity Index, ASA score (American Society of Anesthesiologists), tumor entities, preoperative tumor stage, and preoperative lymph node stage. Additional primary endpoints included surgery duration, blood loss, duration of postoperative ICU-stay, total hospital stay, anastomotic insufficiency, pneumonia, re-operation rates, postoperative resection status, 30-day morbidity, and percentage rates of severe postoperative complications (Clavien-Dindo > 3b). Secondary endpoints included mean subjective pain NRS levels during rest and stress on the first, third and fifth postoperative days (POD), cumulative morphine milligram equivalents (MME), as well as MME normalized by patient weight and duration of stay. 

### 2.6. Statistical Analysis

Data were analyzed using SPSS Version 29.0 (IBM Corp. Released 2022. IBM SPSS Statistics for Windows, Version 29.0, IBM Corp., Armonk, NY, USA) and R Version 4.20 (R Foundation for Statistical Computing, Wirtschaftsuniversität Wien, Vienna, Austria). Levels of categorical and ordinal variables are displayed as percentages, except for NRS-pain values and postoperative complications, which are summarized by medians and interquartile ranges. Continuous variables and durations in days are summarized by medians and interquartile ranges. One patient, who died of sepsis on the fourth postoperative day, was excluded from the analyses of NRS-pain POD 5 and time until bowel movement. Otherwise, there were no missing data. Significance testing was conducted in R employing Fisher’s exact test for categorical variables and Wilcoxon–Mann–Whitney test for all other variables with all *p*-values two sided, and a local significance level of 0.05; unadjusted *p*-values are reported. 

## 3. Results

Our cohort of 168 patients was homogeneous in terms of baseline characteristics. As seen in Table 1, demographic factors, comorbidities (ASA score and Charlson Comorbidity Index), pre-therapeutic tumor stages, and neoadjuvant therapies are comparable between groups.

**Figure 1:**
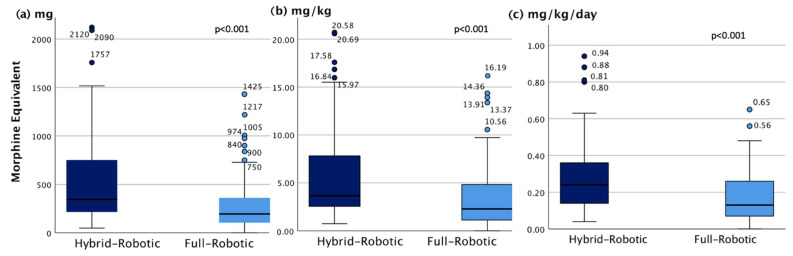
Box-plot chart presenting the Morphine Equivalent Dose in hybrid-RAMIE and full-RAMIE: (**a**) MME, (**b**) MME/kg bw, (**c**) MME/kg/day.

**Figure 2:**
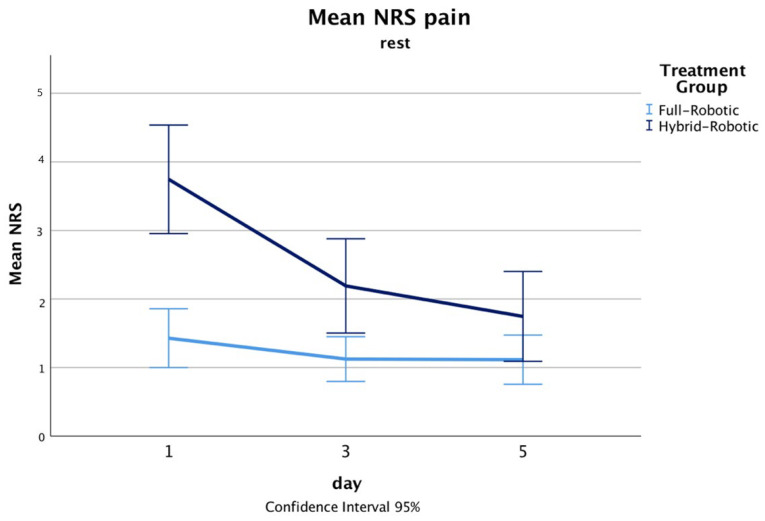
Line chart displaying mean NRS-pain scores at rest on the first, third, and fifth postoperative days.

**Figure 3:**
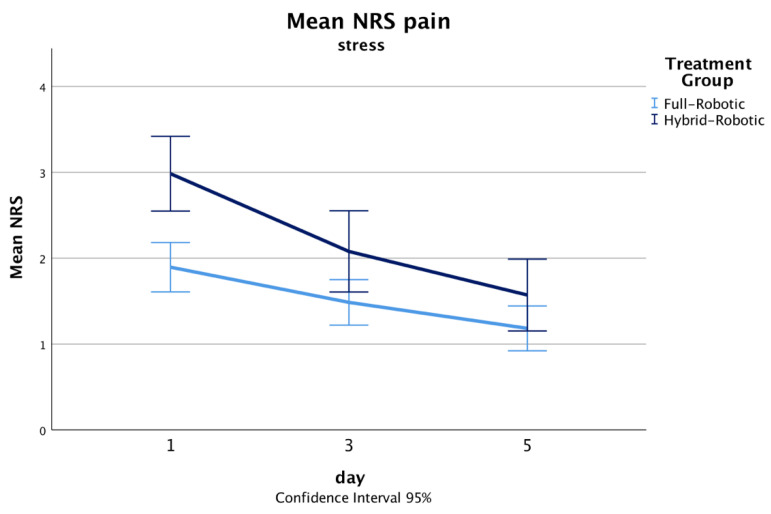
Line chart displaying mean NRS pains scores at stress on the first, third, and fifth postoperative days.

The total operating time was significantly shorter in the full-RAMIE group (median 410, range 367–470) compared with the hybrid-RAMIE group (median 494, range 409–593, *p* < 0.001). While the median blood loss between groups was similar, the upper quartile was substantially lower in the full-RAMIE group (125 mL vs. 300 mL Q3 upper quartile, Table 2). There were no intraoperative conversions and oncological results were similar between groups with a R0 resection rate of about 97%. Median postoperative opioid consumption (Figure 1) was significantly lower in the full-RAMIE group (median 2.28, range 1.12–4.83 MME/kg bw vs. hybrid median 3.66, range 2.54–7.83, *p* < 0.001) (Table 3). Postoperative defecation was similar between groups, and there is no evidence for opioid-adverse events. In addition, mean pain NRS scores were reduced on the first, and third postoperative days, and comparable on the fifth (Figure 2 and Figure 3). 

There was a tendency for fewer severe complications (Clavien-Dindo > 3b) to be registered postoperatively in the full-RAMIE group (hybrid 14.3% vs. full 8.5%; *p* = 0.304). Similarly, regarding anastomotic insufficiencies, there was a trend in favor of the full-RAMIE (hybrid 22.2% vs. full 14.2%; *p* = 0.21). More importantly, we observed significantly fewer pneumonias in the full-RAMIE group (hybrid 20.6% vs. full 7.6%; *p* = 0.017). While total postoperative stays were the same for both patient groups, intensive care stays were significantly shorter in the full-RAMIE group (median 2, range 1–3 vs. median 3, range 2–6; *p* < 0.001). One patient in the full-RAMIE group died within 30 days of surgery due to sepsis (Table 3).

## 4. Discussion

Our results add to the growing body of evidence that robotic-assisted procedures provide better outcomes. The findings highlight the clinical superiority of the full-RAMIE approach both in terms of better surgical outcome and optimized patient care. This is most visible when comparing the risks of postoperative complications such as pneumonia and anastomotic insufficiencies, which were higher or significantly higher among patients who had undergone the hybrid-RAMIE. Conversely, the full-RAMIE group reported less pain, and fewer complications, and experienced shorter ICU stays. We attribute all of these outcomes to the differences in surgical techniques, namely the full-robotic abdominal approach.

Esophagectomy as a procedure is associated with relatively high levels of experienced pain, which may be worsened by excessive manipulation of the abdominal wall. Specifically, we believe that the remote center in the full-RAMIE group leads to reduced trocar movements when compared to the hybrid-RAMIE, and therefore to less manipulation of the abdominal wall during surgery. To this end, between the abdominal and thoracic phases, the abdominal pain appears to have a larger relevance on overall disease-specific pain experienced by the patient—a crucial aspect that would benefit from further evaluation in a randomized control trial. 

Using the robotic system in both abdominal and thoracic phases significantly reduced the median operating time and upper quartile blood loss. We acknowledge that intraoperative parameters such as blood loss and operating time are influenced by surgeon and operating team proficiency, and therefore represent a learning curve that shows consistent improvement overtime. Although there is a significant learning curve associated with RAMIE, structured proctoring learning curves tend to be reached faster and have been shown to plateau after around 22–25 cases [24,28,33,34]. While we acknowledge that some results such as surgery time may be a result of systematic standardization and team experience, the quality of the surgery outcomes—especially pain levels and complications—are independent of any potential learning curves and as such can be imminently linked to the benefits of the full-robotic technique. 

Anastomotic insufficiencies remain the most common postoperative complications [12], and while our results are no exception to this, the full-RAMIE group had relatively fewer incidences than the hybrid group. Although the benefit of a generalized, prophylactic EndoVAC therapy has not yet been established, its use has been standard procedure at our clinic as a way to proactively prevent and detect anastomotic insufficiencies [35]. This could also have had an impact on the overall severity and explain our significantly lower rate when compared to other studies. In both surgical techniques, the thoracic phases were robotic-assisted. Although more studies are needed to determine significance, we attribute the relative reduction to decreased surgical trauma experienced by patients. Minimally invasive surgery has been shown to cause less surgical trauma and immunologic stress, and it is in light of the latter that we observe our low postoperative complication rate [36]. Surgical trauma in both phases, but here specifically in the abdominal phase, directly influences recovery and healing after surgery. 

This reduction in surgical trauma is also reflected in NRS pain scores, as is the need for supplemental opioid analgesics. In both groups, subjective pain levels were relatively low at rest and during activity, which shows effective multimodal pain therapy in both groups. Moreover, there is no evidence for opioid-related adverse outcomes in our patient collective. With multimodal analgesics, pain is expected to be comparable between groups; however, the full-RAMIE group required fewer opioid analgesics than the hybrid-RAMIE group. The full-RAMIE group not only had significantly less pain but as a result but also consumed significantly less morphine-equivalent doses. The significant difference in opioid consumption MME/kg bw/day shows that full-RAMIE patients had less breakthrough pain. This could be due to differences in abdominal insufflation and intraoperative intra-abdominal pressures and manipulation of the abdominal wall during surgery. In laparoscopic abdominal procedures an intraabdominal pressure between 12–15 mmHg is required for adequate visualization. In comparison, robotic assistance allows for intra-abdominal pressure ranging from 8–10 mmHg. Additionally, the robotic trocars hold up and stabilize the abdominal wall with the remote center mechanism, which substantially reduces abdominal wall manipulation and irritation. 

With less pressure on the diaphragm and less abdominal wall irritation, the patients in the full-RAMIE group experienced less pain. Reduced pain could also help explain why the full-RAMIE group had a significantly lower incidence of pneumonia, as intense pain results in ineffective breathing, therefore potentially influencing the risk of lung infection [37]. Not only did we observe fewer postoperative complications and less pain, but the full-RAMIE group also subsequently had a faster recovery and spent less time in the ICU when compared to the hybrid-RAMIE group. To this end, we postulate that less manipulation of the abdominal wall could account for less surgical trauma, postoperative pain, complications, and recovery time. Therefore, the full-RAMIE minimizes patients’ perioperative morbidity and risk.

This study derives its strength from a robust retrospective data collection, which means biases, such as confounders and treatment bias are limited. All data used in the study are routine parameters that were collected for every patient and are independent of the treatment method or individual patient care. Additionally, each patient group received the most technically advanced robotic procedure available at the time of treatment, making a placebo effect between the groups nonexistent. Conversely, selection bias could affect results given the Muenster University Hospital’s national reputation as a high-volume esophagectomy center, which means that some patients could have elected to have their surgery performed here. In addition, information bias could have existed due to missing data and the inherent limitations of routine perioperative patient care management documentation. Our hospital has a quality-management team who continuously monitors medical record consistency and accuracy. Lastly, we acknowledge a time period bias as the procedures were not conducted simultaneously but rather consequently.

Other study limitations include the single-institution study design. While minimally invasive techniques are well established, there is currently only one randomized control trial evaluating RAMIE surgery, and no randomly controlled trials evaluating the differences between various RAMIE techniques [10]. Furthermore, the surgical techniques used (hybrid or full) and terminology applied differ significantly among healthcare providers and across clinical settings. Although structured training has been outlined, a universal standard does not yet exist [34]. This limits reproducibility and transferability to other institutions. Due to the popularity of the Ivor Lewis RAMIE, demand is increasing, and more centers have started offering the procedure, even without proctor-led training. Due to a lack of standardization of training, multiple surgery techniques have evolved, which makes comparison between centers difficult, and further highlights the need for establishing an ideal, universally accepted “gold standard” surgical technique. Under the status quo, patient perioperative risk depends on technique and individual surgical experience.

Further randomized control trial studies comparing full-RAMIE to full-MIE laparoscopic approaches (such as the ROBOT-2 trial) are needed [38]. Additionally, a random control trial comparing hybrid- and full-RAMIE techniques should be performed. While our study does not consider in-depth cost analysis for both RAMIE procedures, the hybrid-RAMIE has higher costs associated with it. By using both robotic and laparoscopic technologies in the hybrid-RAMIE, an additional laparoscopic surgical set, camera, tower, and surgical dissection/stapling systems are needed. These requirements influence not only operating room ergonomics but also hospital economics. Likewise, a quicker recovery, less analgesics, and fewer complications seen in the full-RAMIE have a major impact on hospital economics.

## 5. Conclusions

Our results show that the full-RAMIE is superior to the hybrid-RAMIE for short-term postoperative outcomes and morbidity. While the end anastomosis techniques are identical, we postulate that less manipulation of the abdominal wall in the full-RAMIE group results in less pain and surgical trauma, and therefore a lower incidence of complications. With the additional robotic-assisted abdominal phase in the full-RAMIE group, we see significantly fewer pneumonias, a faster ICU recovery and reportedly less pain. Minimally invasive surgery allows patients to recover more quickly due to less surgical trauma. This represents a potential impact minimally invasive procedures could have on future patient outcomes, if applied on an even greater scale. To this end, RAMIE is also a more patient-centered approach with the potential of improving patients’ immediate outcomes. The enhanced technical aspects in the full-RAMIE procedure allow for a more streamlined, accurate and standardized surgery, which affects disease management as much as it does hospital economics. The accumulative surgeon experience seen in the full-RAMIE also results in shorter operating times. Coupled with meticulous lymph node resection capabilities, the full-RAMIE approach represents a noticeable and permeable surgical–oncological advancement for future patients with esophageal cancer. Ultimately, our findings underscore the relevance of continued research into, and advancement of, full robotic-assisted procedures in the quest for improving surgical outcomes, reducing pain, and establishing a gold standard surgical technique.

## Figures and Tables

**Table 1:** Baseline characteristics (n = 168).

	Full-RAMIE (n = 105) n (%)	Hybrid-RAMIE (n = 63) n (%)	*p*-Value
Age, years			*p* = 0.05
<65	46 (43.8)	39 (61.9)	
65–75	41 (39.1)	14 (22.2)	
>75	18 (17.1)	10 (15.9)	
Sex			*p* = 1
F	17 (16)	10 (15.9)	
M	88 (84)	53 (84.1)	
Ethnicity			
White	105 (100)	63 (100)	
BMI, kg/m^2^			*p* = 0.113
<20	8 (7.6)	2 (3.2)	
20–30	77 (73.3)	41 (65.1)	
>30	20 (19)	20 (31.7)	
ASA score			*p* = 0.314
1	3 (2.9)	2 (3.2)	
2	50 (47.6)	35 (55.6)	
3	52 (49.5)	26 (41.3)	
4	0 (0)	0 (0)	
Type of carcinoma			*p* = 0.513
Adenocarcinoma	87 (82.9)	55 (87.3)	
Squamous cell carcinoma	18 (17.1)	8 (12.7)	
Locations of tumor			*p* = 0.555
Upper third	1 (1.0)	0 (0)	
Middle third	12 (11.4)	4 (6.3)	
Gastroesophageal junction	92 (87.6)	59 (93.7)	
Neoadjuvant therapy			*p* = 0.269
Chemotherapy	31 (29.5)	24 (38.1)	
Chemoradiotherapy	65 (61.9)	31 (49.2)	
None	9 (8.6)	8 (12.7)	
Charlson Comorbidity Index			*p* = 0.07
1	7 (6.7)	0 (0)	
2	8 (7.6)	1 (1.6)	
3	21 (20.0)	11(17.5)	
4	24 (22.9)	13 (20.6)	
5	14 (13.3)	19 (30.2)	
6	13 (12.4)	10 (15.9)	
7	8 (7.6)	4 (6.3)	
8	5 (4.8)	3 (4.8)	
9	4 (3.8)	2 (3.2)	
10	0 (0)	0 (0)	
11	1 (1.5)	0 (0)	
pretherapeutic T-status			*p* = 0.645
T1	10 (9.5)	7 (11.1)	
T2	20 (19.0)	13 (20.6)	
T3	73 (69.5)	42 (66.7)	
T4	2 (1.9)	1 (1.6)	
pretherapeutic N-status			*p* < 0.01
N0	57 (54)	16 (25)	
N+	48 (46)	47 (74)	

BMI: body mass index; ASA: American Society of Anesthesiologists.

**Table 2:** Intraoperative and pathological statistics (n = 168).

	Full-RAMIE (n = 105)	Hybrid-RAMIE (n = 63)	*p*-Values
Operating time, min *	410 (367, 470)	494 (409, 593)	*p* < 0.001
Blood loss, mL *	0 (0, 125)	0 (0, 300)	*p* = 0.5
Conversions [n (%)]	0 (0)	0 (0)	
Conversion thorax	0 (0)	NA	
Conversion abdomen	0 (0)	0 (0)	
Pathological T-status [n (%)]			*p* = 0.8
T0	26 (24.8)	14 (22.2)	
T1	12 (11.4)	12 (19.1)	
T2	20 (19)	6 (9.5)	
T3	47 (44.8)	31 (49.2)	
Pathological N-status [n (%)]			*p* = 0.848
N0	57 (54.3)	35 (55.6)	
N1	23 (21.9)	9 (14.3)	
N2	16 (15.2)	13 (20.6)	
N3	9 (8.6)	6 (9.5)	
Radicality of surgery [n (%)]			*p* = 1.00
R0	102 (97.1)	61 (96.8)	
R1	3 (2.9)	2 (3.2)	

* median (interquartile range).

**Table 3:** Postoperative statistics (n = 168).

	Full-RAMIE (n = 105)	Hybrid-RAMIE (n = 63)	*p*-Values
Morphine-equivalent-dose total, mg *	194 (105, 360)	345 (215, 750.5)	*p* < 0.001
Morphine-equivalent-dose total/kg bw, mg/kg *	2.28 (1.12, 4.83)	3.66 (2.54, 7.83)	*p* < 0.001
Morphine-equivalent-dose total/kg bw/days hospital stay, mg/kg/days *	0.13 (0.07, 0.26)	0.24 (0.14, 0.36)	*p* < 0.001
NRS pain POD 1 *			
rest	0 (0, 1)	2 (1, 3)	*p* < 0.001
stress	2 (1, 3)	3 (2, 4)	*p* < 0.001
NRS pain POD 3 *			
rest	0 (0, 1)	1 (0, 2)	*p* = 0.006
stress	1 (0, 2)	2 (1, 3)	*p* = 0.058
NRS pain POD 5 *			
rest	0 (0, 1)	0 (0, 1)	*p* = 0.14
stress	1 (0, 2)	1 (0, 3)	*p* = 0.152
Complications, MCDC *	0 (0, 3)	2 (0, 3)	*p* = 0.004
Severe complication, MCDC ≤ 3b [n (%)]	9 (8.5)	9 (14.3)	*p* = 0.304
Pneumonia [n (%)]	8 (7.6)	13 (20.6)	*p* = 0.017
Anastomotic leakage ^a^ [n (%)]	15 (14.2)	14 (22.2)	*p* = 0.21
Type I (conservative)	0 (0)	0 (0)	
Type II (nonsurgical intervention)	13 (12.3)	10 (15.9)	
Type III (surgical intervention)	2 (1.9)	4 (6.3)	
Reoperations [n (%)]	5 (4.7)	7 (11.1)	*p* = 0.134
Hospital stay, days *	18 (14, 25)	19 (14, 27)	*p* = 0.479
ICU stay, days *	2 (1, 3)	3 (2, 6)	*p* < 0.001
Epidural anesthesia, days *	5 (5, 6)	5 (5, 6)	*p* = 0.007
Time until bowel movement, days *	5 (4, 7)	4 (3, 6)	*p* < 0.001
30-Day mortality [n (%)]	1 (0.9)	0 (0)	*p* = 1.00

* median (interquartile range). bw, body weight; POD, Postoperative Day; MCDC, modified Clavien Dindo classification; ICU, intensive care unit. ^a^ Complications graded according to ECCG [12].

## Data Availability

The data presented in this study are available on request from the corresponding author. The data are not publicly available.
